# Smooth muscle cell-specific knockout of neuropilin-1 impairs postnatal lung development and pathological vascular smooth muscle cell accumulation

**DOI:** 10.1152/ajpcell.00405.2018

**Published:** 2019-01-16

**Authors:** Marwa Mahmoud, Ian M. Evans, Vedanta Mehta, Caroline Pellet-Many, Ketevan Paliashvili, Ian Zachary

**Affiliations:** Centre for Cardiovascular Biology and Medicine, BHF Laboratories, Division of Medicine, University College London, London, United Kingdom

**Keywords:** alveolar myofibroblast, neointima, neuropilin-1, SMC

## Abstract

Neuropilin 1 (NRP1) is important for neuronal and cardiovascular development due to its role in conveying class 3 semaphorin and vascular endothelial growth factor signaling, respectively. NRP1 is expressed in smooth muscle cells (SMCs) and mediates their migration and proliferation in cell culture and is implicated in pathological SMC remodeling in vivo. To address the importance of *Nrp1* for SMC function during development, we generated conditional inducible *Nrp1* SMC-specific knockout mice. Induction of early postnatal SMC-specific *Nrp1* knockout led to pulmonary hemorrhage associated with defects in alveogenesis and revealed a specific requirement for *Nrp1* in myofibroblast recruitment to the alveolar septae and PDGF-AA-induced migration in vitro. Furthermore, SMC-specific *Nrp1* knockout inhibited PDGF-BB-stimulated SMC outgrowth ex vivo in aortic ring assays and reduced pathological arterial neointima formation in vivo. In contrast, we observed little significant effect of SMC-specific *Nrp1* knockout on neonatal retinal vascularization. Our results point to a requirement of *Nrp1* in vascular smooth muscle and myofibroblast function in vivo, which may have relevance for postnatal lung development and for pathologies characterized by excessive SMC and/or myofibroblast proliferation.

## INTRODUCTION

Neuropilin-1 (NRP1) is a transmembrane glycoprotein receptor essential for both vascular and neuronal development due to its role in mediating vascular endothelial growth factor (VEGF) and class 3 semaphorin signaling, respectively (14, 16, 25, 36). *Nrp1*-null mice are embryonic lethal between embryonic day (E) 10.5 and E14.5, dependent on genetic background, and display a spectrum of cardiovascular and neuronal defects (21, 23).

Several studies have reported NRP1 expression in smooth muscle cells (SMCs) and revealed an important role for NRP1 in vascular SMC migration (13, 27, 30, 35). NRP1 expression has also been reported in SMCs in vivo in large vessels (20) and targeted knockdown of NRP1 in vivo using small hairpin inhibitory RNA-inhibited neointima formation induced by rat carotid artery endovascular injury (32). Mice with constitutive SMC-specific loss of *Nrp1* were viable, displaying no overt early postnatal phenotype but exhibiting a late-onset defect in gastrointestinal motility due to loss of visceral SMC contractility (46). The lack of an overt phenotype in constitutively SMC-specific *Nrp1*-null mice could be indicative of genetic redundancy, as reported for other constitutive knockout mice (5, 17, 42). NRP1 shares a similar domain structure and 44% amino acid sequence homology with neuropilin-2 (NRP2), which is also expressed in vascular smooth muscle cells (10, 30–32). Mice lacking *Nrp1* and *Nrp2* die earlier in embryogenesis (E8.5) and display more severe defects in angiogenesis compared with single knockouts (39). Moreover, *Nrp2* compensates for loss of semaphorin binding to NRP1 in *Nrp1^Sema/Sema^* mice (15). These data suggest a partial genetic redundancy between *Nrp1* and *Nrp2*, which could explain the lack of an overt phenotype in constitutive SMC-specific *Nrp1*-null mice.

To circumvent genetic redundancy effects due to possible compensatory upregulation of *Nrp2* expression, we generated a conditional and inducible *smMHC-Cre^ERT2^* transgenic line to allow controlled *Nrp1* knockout in SMCs when crossed to *Nrp1* floxed mice, by administration of tamoxifen during development. Our results revealed a specific requirement for *Nrp1* in myogenic cell lineages during pulmonary microvascular expansion and alveogenesis. *Nrp1* was also necessary for pathological neointima formation in vivo, for PDGF-BB-induced SMC outgrowth ex vivo in aortic rings, and for PDGF-AA-induced pulmonary myofibroblast migration in cultured cells.

## MATERIALS AND METHODS

### 

#### Generation and characterization of mice.

All procedures involving mice were conducted under a UK Home Office license and the approval of the University College London local ethics committee in accordance with the Animal Care and Ethics Guidelines and the 1986 United Kingdom Home Office Animals (Scientific Procedures) Act.

Inducible SMC-specific *Nrp1* knockout mice were generated by crossing *Nrp1^floxed^; R26R* mice to *smMHC-Cre^ERT2^* transgenic mice, on a C57BL/6 background. Genotypes of progeny were identified by PCR using the primers listed in Table 1. To induce knockout in neonates, mice were given two subcutaneous 0.5 mg tamoxifen injections at P1–2 and P3–4. For adults (>8 wk), 5 consecutive 1-mg tamoxifen injections were administered intraperitoneally over 5 days.

**Table 1.** Details of genotyping primers

Gene Targeted	Genotyping Primer	Sequence (5′–3′)
Floxed *Nrp1* allele	flNrp1_F1	CAA TGA CAC TGA CCA GGC TTA TCA TC
	flNrp1_R1	GAT TTT TAT GGT CCC GCC ACA TTT GTC
Recombined *Nrp1* allele	rNrp1_F2	AGG CCA ATC AAA GTC CTG AAA GAC AGT CCC
	rNrp1_R2	TCT GCA GAT CAT GTA TAC TGG TGA CCC ACA
*smMHC-Cre^ERT2^* transgene	SMWT1	TGA CCC CAT CTC TTC ACT CC
	SMWT2	AAC TCC ACG ACC ACC TCA TC
	PhCREAS1	AGT CCC TCA CAT CCT CAG GTT
*LacZ* allele	LacZ1	AAA GTC GCT CTG AGT TGT TAT
	LacZ2	GGA GCG GGA GAA ATG GAT ATG
	LacZ3	GCG AAG AGT TTG TCC TCA ACC

*Nrp1*, neuropilin 1.

#### Histology.

Tissue was fixed in HistoChoice tissue fixative (Sigma) or zinc fixative (BD PharMingen) overnight and then processed to paraffin and cut into 10-µm sections, except for the retinas, which were fixed in 4% paraformaldehyde and stained as whole-mounts. Samples were subjected to hematoxylin-eosin histological staining, immunohistochemistry, or immunofluorescence using the antibodies and conditions listed in Table 2. X-Gal staining was performed using the LacZ Detection Kit by InvivoGen (no. rep-lz-t), according to the manufacturer’s instructions. Alveolar hemorrhaging was identified by the incidence of hemosiderin-laden macrophages, as previously documented (1).

**Table 2.** Antibodies used for histology

Protein	Antibody Details	Working Dilution	Antigen Retrieval/Special Kit Used
α-SMA	Monoclonal anti-actin, α-smooth muscle, clone 1A4. Sigma no. A2547.	1/100	M.O.M. Kit (Vector Labs no. BMK-2202)
NG2	Anti-NG2 chondroitin sulfate proteoglycan antibody. Millipore no. AB5320.	1/200	Citrate buffer (pH 6.0)
Isolectin B4	DyLight 594 Labeled Griffonia Simplicifolia Lectin I (GSL I) isolectin B4. Vector Labs no. DL-1207.	1/100	
α-SMA	Monoclonal anti-actin, α-smooth muscle-FITC antibody, clone 1A4. Sigma no. F3777.	1/100	
NRP1	Neuropilin 1 antibody. GeneTex no. GTX127947.	1/100	Citrate buffer (pH 6.0)
Ki67	Ki-67 (D3B5) rabbit mAb. Cell Signaling Technology no. 12202.	1/200	Citrate buffer (pH 6.0)

NG2, neural/glial antigen 2; NRP1, neuropilin 1; SMA, smooth muscle actin.

#### Transwell migration assay.

Mouse pulmonary fibroblasts (no. M3300-57, ScienCell) were cultured in poly-l-lysine-coated dishes and treated with 10 ng/ml transforming growth factor-β1 (TGF-β1; no. AF-100-21C, Peprotech) for 48 h to induce differentiation to myofibroblasts. Cells were transfected with either scrambled siRNA or two different siRNAs targeting *Nrp1* (siRNA cat. nos. 70800 and s70802, respectively; Thermo Fisher Scientific) using RNAiMAX transfection reagent ( no. 13778075, Thermo Fisher Scientific). Transfected cells were seeded at a count of ~2.5 × 10^4^ cells/Transwell insert (8.0-μm pore size, no. 353097, Falcon) and left to migrate for 20–24 h in response to either serum-free media or serum-free media with 50 ng/ml PDGF-AA (100-13A, Peprotech). Nonmigrated cells were removed, and Transwell inserts were stained using the REASTAIN Quick-Diff Kit, according to manufacturer’s instructions (no. 102164, Gentaur), and migrated cells were counted under a Leica stereo microscope.

#### Western blotting.

Western blot analysis was performed on transfected myofibroblast cell lysates as described previously (11), using the following primary antibodies: neuropilin-1 (no. ab81321, Abcam), α-smooth muscle actin (α-SMA; no. ab7817, Abcam), and β-actin (no. A2228, Sigma).

#### Aortic ring assay.

Aortas from 8- to 10-wk-old male mice that had been treated with tamoxifen the week before harvest were dissected and cultured as described previously (3, 9) in the presence of 1 µM 4-hydroxytamoxifen overnight, after which rings were incubated in fresh medium-containing cytokines. Cell growth and sprout formation were monitored using the IncuCyte Live Cell Analysis System (Essen BioScience). Assays were stopped 6 days postembedding and subjected to double immunofluorescence staining for endothelial-specific isolectin B4 (no. DL-1207, Vector Laboratories) and α-SMA (no. F3777, Sigma) to visualize vascular sprouts.

#### Perivascular cuff model.

Surgeries were performed as described (29). Briefly, under general anesthesia (using inhaled isoflurane), the femoral artery is exposed, and a polyethylene cuff (with a sagittal opening to allow placement of the artery) is loosely placed around the artery and secured in place with sutures. Sham surgical controls were handled in the same manner, except no cuff placement was performed. All mice were treated with tamoxifen the week before the surgery. Twenty-one days following the surgery, the mice were euthanized by overdose of CO_2_, followed by cervical dislocation, and the cuffed femoral arteries and sham controls were harvested for analysis.

#### Measurements and statistical analyses.

Pulmonary hemorrhaging was quantified by blinded measurements of the percentage of hemorrhaging per field of view from at least three different sections per sample. Statistical significance was calculated using the Kruskal-Wallis H test with post hoc Dunn’s multiple comparison test. Quantification of α-SMA expression and numbers of presumptive septal tips with α-SMA-expressing myofibroblasts was determined using the ImageJ Color Threshold function or the ImageJ Cell Counter plugin, respectively. Data were collected and analyzed blindly from at least three different sections/fields of view per sample.

Outgrowth areas in aortic ring assays were quantified as described (8), using the ImageJ Color Threshold function (with background subtraction).

Unless specified otherwise, all data are presented as means ± SE, and statistical significance was calculated using the two-tailed unpaired Student’s *t*-test, except for the perivascular cuff intima/media ratio data, which were analyzed using the one-tailed unpaired *t*-test, and the myofibroblast Transwell migration data, which were analyzed using two-way ANOVA with Bonferroni post test.

## RESULTS AND DISCUSSION

### 

#### SMC-specific knockout of Nrp1 in early postnatal development causes pulmonary hemorrhaging and impaired alveolar development.

**Fig. 1.**
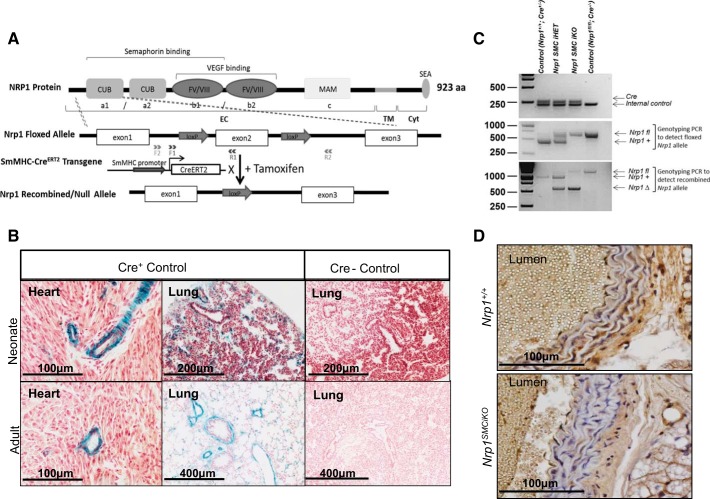
Characterization of *Nrp1* smooth muscle cell (SMC)-specific inducible knockout mice. *A*: floxed *Nrp1* mice have exon-2 flanked by two loxP sites, which undergo Cre-mediated recombination exclusively in SMCs when crossed to the *smMHC-Cre^ERT2^* transgenic mice and given tamoxifen. Location of the floxed *Nrp1* and recombined *Nrp1* PCR primers is depicted by F1 and R1 and F2 and R2, respectively. *B*: X-gal staining showing Cre recombinase activity in *Cre+* and *Cre−* mice. *C*: genotyping PCR used to identify transgenic mice. The recombined *Nrp1* allele is only detected following treatment of the mice with tamoxifen. *D*: immunostaining for NRP1 in the aortae of *Nrp1^SMCiKO^* and *Nrp1^+/+^* mice. NRP1, neuropilin 1; SEA, PDZ-binding domain motif; EC, extracellular domain; MAM, meprin, A5 antigen, and receptor protein phosphatase-μ domain; CUB, complement binding factors C1s/C1r, Uegf, BMP-1 domain; TM, transmembrane domain; Cyt, cytoplasmic domain.

S*mMHC-Cre^ERT2^* mice (44) were bred with *Nrp1^floxed^* mice (14), previously crossed to the *R26R-LacZ* reporter strain (37), to generate *Nrp1^SMCiKO^* mice, allowing for inducible ablation of *Nrp1* in SMCs (Fig. 1*A*). Induction of Cre recombinase activity, monitored by expression of β-galactosidase, following administration of tamoxifen in adult and neonatal *Nrp1^SMCiKO^* mice, was restricted to SMCs in multiple tissues, including the heart and lungs (Fig. 1*B*). *Nrp1* allele recombination and protein knockdown in SMC were confirmed by genotyping PCR (Fig. 1*C*) and immunostaining (Fig. 1*D*).

**Fig. 2.**
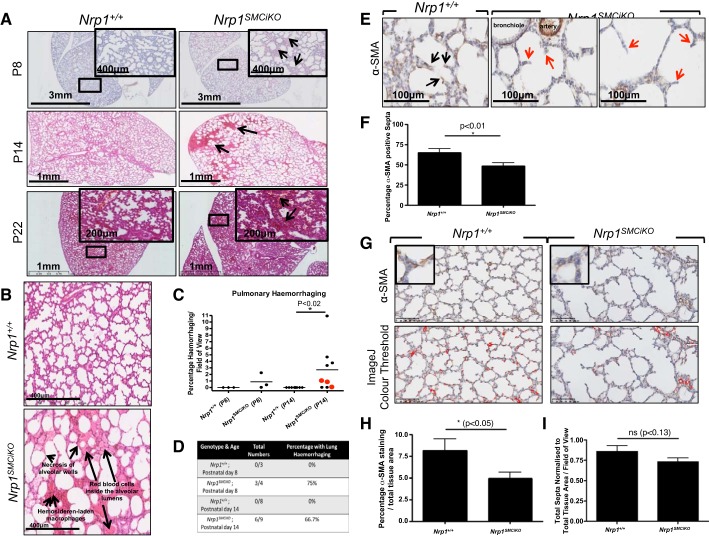
Pulmonary phenotype in *Nrp1^SMCiKO^* mice. *A*: hematoxylin-eosin staining of *Nrp1^+/+^* and *Nrp1^SMCiKO^* lung sections. Arrows indicate pulmonary hemorrhaging, which is detected from P8, increases to P14, and is reduced by P22. *B*: high-magnification image indicating hemorrhaging and alveolar wall necrosis in *Nrp1^SMCiKO^* lung. *C*: quantification of hemorrhaging at P14. *P < 0.02 vs. *Nrp1^+/+^*. Red symbols indicate mice treated with tamoxifen at P3; all other mice were treated at P1. *D*: incidence of pulmonary hemorrhaging in the *Nrp1^SMCiKO^* neonates. *E*: immunostaining of lung sections for α-SMA. Black arrows indicate α-SMA-positive septal tips in *Nrp1^+/+^* alveoli, and red arrows indicate α-SMA-negative septal tips in *Nrp1^SMCiKO^* alveoli. *F*: analyses of the percentage of α-SMA-positive secondary alveolar septal tips at P14; *P < 0.01 vs. *Nrp1^+/+^*. *G*: α-SMA staining was quantified using the ImageJ Color Threshold function; representative images showing detection of α-SMA staining (highlighted in red) by ImageJ are shown. Boxed images are higher-power views showing α-SMA expression in the alveoli. *H*: quantification of α-SMA staining at P14; *P < 0.05 vs. *Nrp1^+/+^*. *I*: quantification of total numbers of septae at P14; *P* = ns vs. *Nrp1^+/+^*. *n* = 4 *Nrp1^+/+^* and *n* = 6 *Nrp1^SMCiKO^*. Experiments were performed on male and female mice. NRP1, neuropilin 1; ns, not significant; P, postnatal day; SMA, smooth muscle actin.

To determine the role of SMC-specific *Nrp1* in early postnatal development, we conditionally ablated *Nrp1* expression using tamoxifen from postnatal day (P)1–2 and monitored the effect of *Nrp1* loss. The *Nrp1^SMCiKO^* neonates appeared viable and healthy compared with their littermate controls; however, internal examination revealed hemorrhaging in the lungs of the *Nrp1^SMCiKO^* neonatal pups from P8 (Fig. 2*A*). Hemorrhaging was generally localized to the lung periphery and characterized by the presence of hemosiderin-laden macrophages, which are an indication of long-term alveolar hemorrhaging (>48 h), and evidence of necrosis of the alveolar walls (Fig. 2*B*). Hemorrhaging was most marked and statistically significant compared with control littermates at P14 (Fig. 2*C*) but had partially resolved by P22. This phenotype was not fully penetrant, with 75% and 66.7% of *Nrp1^SMCiKO^* mutants displaying pulmonary hemorrhaging at P8 and P14, respectively (Fig. 2*D*). Timing of the knockout was critical, as a later induction with tamoxifen at P3–4 led to a much milder hemorrhaging phenotype (red data points in Fig. 2*C*).

**Fig. 3.**
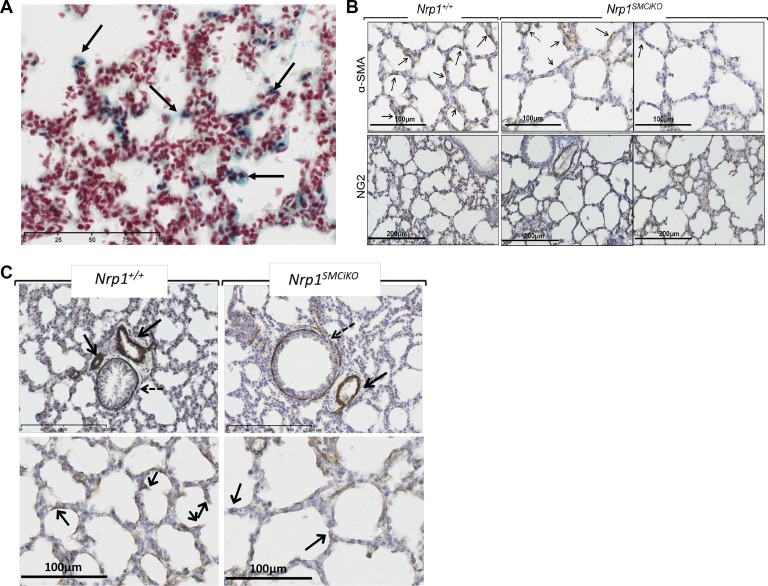
Pulmonary phenotype in the *Nrp1^SMCiKO^* mice. *A*: X-gal expression in P8 lungs showing Cre recombinase expression in cells with locations corresponding to presumptive alveolar myofibroblasts (arrows). *B*: α-SMA and NG2 staining in the lungs of *Nrp1^SMCiKO^* mice and littermate controls. Arrows indicate α-SMA-positive staining in the alveoli, and the dashed arrow points to a small blood vessel (probably a precapillary arteriole). *C*: α-SMA staining of arterioles (arrows) and bronchioles (dashed arrows) in the lungs of *Nrp1^SMCiKO^* mice and littermate controls at P14 (*top*) and α-SMA staining of alveolar capillaries (*bottom*; arrows indicate presumptive septal tips in the lungs of *Nrp1^SMCiKO^* mice and littermate controls at P14). Experiments were performed on male and female mice. NG2, neural/glial antigen 2; NRP1, neuropilin 1; P, postnatal day; SMA, smooth muscle actin.

To determine the underlying cause of the pulmonary hemorrhaging, we examined SMC and myofibroblast coverage of the lung by immunohistochemical staining for α-SMA expression. Whereas SMCs are present in larger blood vessels and muscularized airways, myofibroblasts are specialized cells present on the alveolar septa in the developing postnatal lung and are identified by their expression of α-SMA and their location at septal tips (7, 45). This revealed a loss of alveolar myofibroblasts in the secondary alveolar septae in the *Nrp1^SMCiKO^* mutants (Fig. 2*E*, red arrows), suggesting a defect in the recruitment of alveolar myofibroblasts to the secondary alveolar septae of neonatal mice following *Nrp1* depletion. Quantification of alveolar myofibroblast recruitment, based on their expression of α-SMA and their location at septal tips (22, 45), showed a significant reduction in myofibroblasts associated with septae in *Nrp1^SMCiKO^* mice (Fig. 2*F*). In addition, a significant reduction in overall coverage of α-SMA-positive cells occurred in the alveoli of the *Nrp1^SMCiKO^* mutants (Fig. 2, *G* and *H*). The number of alveolar septae was not significantly different between the *Nrp1^SMCiKO^* mutants and controls when normalized to total tissue area, although a trend towards a reduction was observed (Fig. 2*I*). The expression of Cre recombinase in areas corresponding to the location of alveolar myofibroblasts was confirmed by X-Gal staining (Fig. 3*A*), which demonstrated expression of *smMHC-Cre^ERT2^* and recombination of the *Nrp1* allele in these cells. Recruitment of myofibroblasts to septal tips is important for terminal airway branching and future alveolarization (6, 7), and concomitant with loss of alveolar myofibroblasts in the septae of the *Nrp1^SMCiKO^* mutants (Figs. 2, *E* and *F* and 3*C*), we observed evidence of impaired alveolar development at P14, as indicated by fewer and larger alveoli in the *Nrp1^SMCiKO^* mutants versus littermate controls (Fig. 2, *A* and *G*). No significant changes in expression of the pericyte-specific marker neural/glial antigen 2 (NG2) were observed in the lungs of P14 *Nrp1^SMCiKO^* neonates (Fig. 3*B*), suggesting that inducible *Nrp1* knockout did not impact pericyte recruitment to the vasculature, consistent with the reported lack of expression of *smMHC* in pericytes (41, 43). The reduction in α-SMA expression was also limited to the alveolar septae and capillaries, whereas α-SMA staining of larger arteries and arterioles did not appear to be affected (Fig. 3, *B* and *C*). Given the important role of myofibroblasts in alveolarization during the rapid early postnatal growth and remodeling of the lung, we hypothesize that the observed hemorrhaging resulting from SMC-specific *Nrp1* knockout may be due to defective alveolarization consequent upon impaired myofibroblast recruitment, which in turn results in capillary loss and a reduced capacity of the developing lung to accommodate increased blood flow. Additionally, an impairment of capillary stability due to reduced smooth muscle cell recruitment to capillaries and small vessels may also contribute to the hemorrhaging phenotype in *Nrp1^SMCiKO^* neonates. Further work is required to establish more precisely the causal links between defective myofibroblast recruitment and hemorrhaging in the lungs of *Nrp1^SMCiKO^* mice.

#### PDGF stimulation of pulmonary myofibroblast migration is inhibited following Nrp1 knockout.

**Fig. 4.**
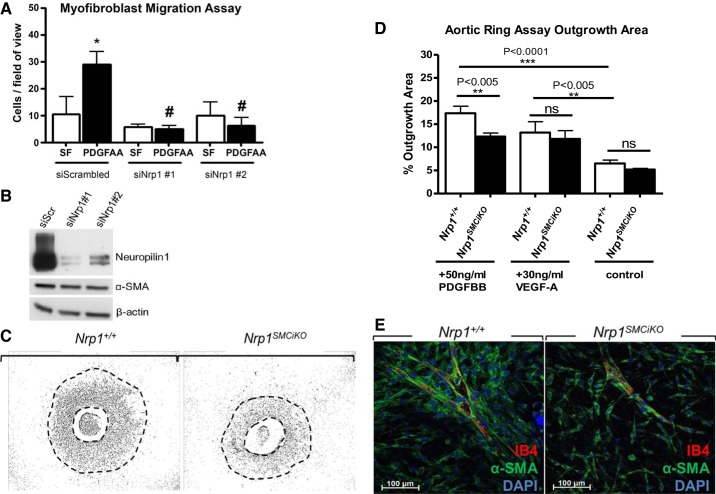
Nrp1 is required for PDGF-induced migration of myofibroblasts and SMCs. *A*: pulmonary myofibroblast Transwell migration assays in response to PDGF-AA compared with serum-free (SF; siScrambled, *n* = 3; siNrp1 #1, *n* = 3; siNrp1 #2, *n* = 3). *P < 0.05 vs. siScr SF and # *P* < 0.01 vs. siScr PDGF-AA. *B*: Western blotting for Nrp1, α-SMA, and the housekeeping control β-actin in transfected pulmonary myofibroblast cell lysates. *C*: SMC-Nrp1 is required for PDGF-BB-induced migration and proliferation in the aortic ring assay. Representative aortic rings; dashed lines highlight the boundaries of areas used for quantitation. *D*: outgrowth assays in *Nrp1^+/+^* and *Nrp1^SMCiKO^* aortic rings treated with PDGF-BB (*n* = 5 *Nrp1^+/+^*, and *n* = 6 *Nrp1^SMCiKO^*) or VEGF-A (*n* = 4 *Nrp1^+/+^*, and *n* = 2 *Nrp1^SMCiKO^*). *E*: immunofluorescent staining of endothelial cells (IB4, red) and vascular smooth muscle cells (α-SMA, green) in aortic ring outgrowths. Aortic ring assays were performed using male mice. NRP1, neuropilin 1; ns, not significant; SMA, smooth muscle actin; SMC, smooth muscle cell.

Platelet-derived growth factor subunit A (PDGF-A) signaling is required for pulmonary myofibroblast differentiation and migration, as evidenced by the absence of myofibroblasts from the primary alveolar septa in PDGF-A-null mice, resulting in severe alveolarization defects (7, 22, 26). To determine the effect of *Nrp1* loss on PDGF-induced myofibroblast migration, we performed in vitro Transwell migration assays. Pulmonary fibroblasts isolated from isolated from postnatal day 2 C57BL/6 mouse lung were differentiated into myofibroblasts in vitro based on cell plating density and treatment with TGF-β1, as previously described (28), and PDGF-AA-induced myofibroblast migration was then measured after transfection with either scrambled siRNA or two different *Nrp1*-specific siRNAs. The results showed a significant reduction in the migration of NRP1-depleted myofibroblasts in response to PDGF-AA (Fig. 4*A*). The efficiency of Nrp1 protein knockout by siRNA was confirmed by Western blotting (Fig. 4*B*). Nrp1 siRNA knockout in myofibroblasts caused no observable effects on viability (results not shown) or α-SMA expression levels compared with control myofibroblasts transfected with scrambled siRNA (Fig. 4*B*).

#### PDGF stimulation of SMC migration is inhibited following Nrp1 knockout.

NRP1 is implicated in the regulation of PDGF signaling required for migration and proliferation in mesenchymal stem cells (4) and vascular SMCs (30, 32). The effect of *Nrp1* loss on PDGF-induced myogenic cell proliferation, migration, and recruitment to neovascular sprouts was determined ex vivo in aortic ring assays. A marked reduction in SMC outgrowth in response to PDGF-BB was observed in the *Nrp1^SMCiKO^* aortic rings versus wild-type controls following treatment with tamoxifen (Fig. 4, *C* and *D*). Although SMCs appeared to be recruited normally to the developing vascular sprouts in this model (Fig. 4*E*), the sprouts in the *Nrp1^SMCiKO^* aortic rings exhibited reduced branching compared with the wild-type aortic rings. Since prevention of VEGF binding to NRP1 blocks endothelial vascular sprouting in aortic rings (12), we also examined the effect of SMC-specific *Nrp1* knockout on the VEGF response. In contrast to the impaired response to PDGF-BB, VEGF-induced aortic ring vessel sprouting was not reduced in *Nrp1^SMCiKO^* aortic rings, indicating that the effect of *Nrp1* ablation was specific for SMCs and did not impact indirectly on endothelial outgrowth.

#### Retinal vascular development is largely unaffected by SMC-specific loss of Nrp1.

**Fig. 5.**
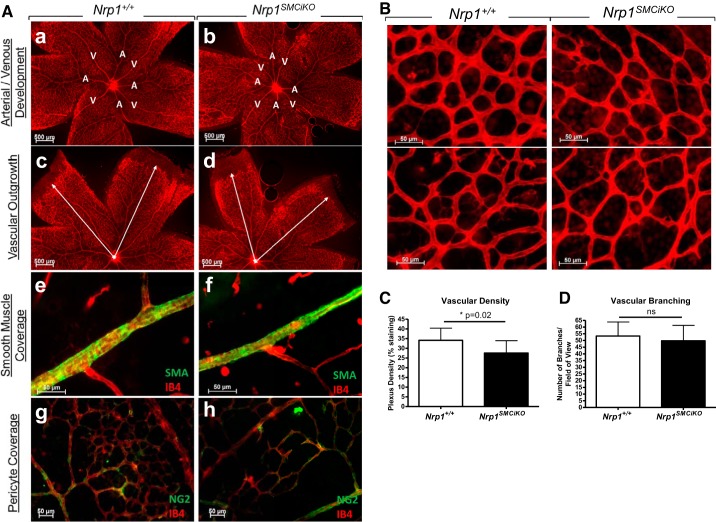
SMC-specific Nrp1 loss results in a mild reduction in retinal vascular density, but retinal vascular development is largely unaffected. *A*: retinal vascular development showing arterial/venous differentiation (*a* and *b*), vascular outgrowth (*c* and *d*), SMC coverage (*e* and *f*), and pericyte coverage (*g* and *h*) in the *Nrp1^SMCiKO^* mutants (*b*, *d*, *f* and *h*) vs. controls (*a*, *c*, *e*, and *g*) at P8. *B*: representative images from P8 wild-type and *Nrp1^SMCiKO^* retinas stained with endothelial isolectin B4. *C*: quantification of vascular density at P8. *P = 0.02 vs. *Nrp1^+/+^*. *D*: quantification of vascular branching at P8; *P* = ns vs. *Nrp1^+/+^*. *n* = 3 *Nrp1^+/+^*, and *n* = 3 *Nrp1^SMCiKO^*. Experiments were performed on male and female mice. NRP1, neuropilin 1; ns, not significant; SMA, smooth muscle actin; SMC, smooth muscle cell.

The impact of SMC-specific loss of *Nrp1* in developmental angiogenesis was further investigated in the neonatal mouse retina, in which the vasculature develops radially from the central optic nerve from P0 and matures into a hierarchical network of arteries, veins, and interconnecting capillaries (38). Following ablation of *Nrp1* expression in *Nrp1^SMCiKO^* neonates from P1, we could not detect any delay in vascularization, defects in arterial/venous differentiation, or an effect on SMC/pericyte coverage of the retinal vessels in the *Nrp1^SMCiKO^* neonates versus their littermate controls (Fig. 5*A*). However, a small, statistically significant reduction in vascular density due to smaller capillary diameters was detected in *Nrp1^SMCiKO^* retinas, whereas vascular branching appeared unaffected (Fig. 5, *B*–*D*). Since *Nrp1* loss in our model is restricted to SMCs and myofibroblasts, whereas pericytes are unaffected, it is possible that the mild retinal phenotype observed results from a restricted role of myofibroblast-mediated vascular remodeling in retinal vascularization.

#### Pathological neointima formation is attenuated following SMC-Nrp1 knockout.

**Fig. 6.**
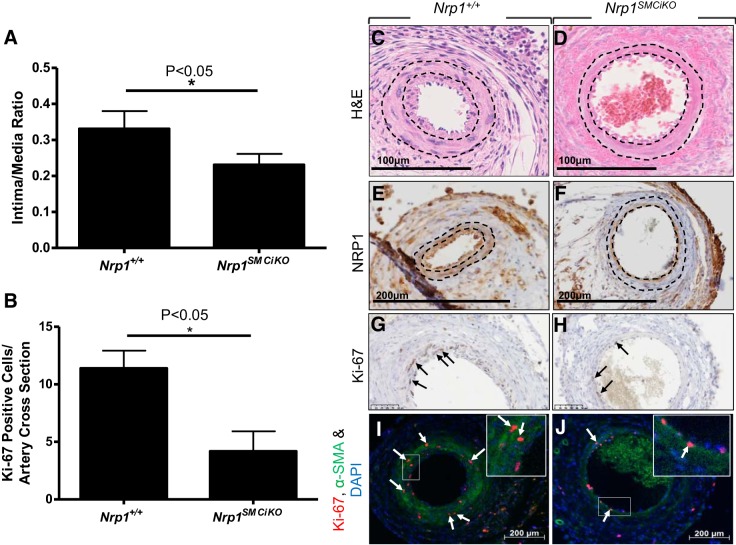
Neointima formation is reduced following SMC-*Nrp1* knockout. *A*: intima/media ratios of cuffed femoral arteries from tamoxifen treated male *Nrp1^+/+^* (*n* = 9) and *Nrp1^SMCiKO^* (*n* = 10) mice. *B*: quantification of *K*_i_-67-positive proliferating cells at 21 days postsurgery. *P < 0.05 vs. tamoxifen-treated *Nrp1^+/+^* (*n* = 5 *Nrp1^+/+^*, and *n* = 5 *Nrp1^SMCiKO^*). *C*–*J*: representative sections of cuffed arteries were H&E-stained (*C* and *D*) or immunostained for NRP1 (*E* and *F*), *K*_i_-67 (*G* and *H*), or costained for *K*_i_-67 and α-SMA (*I* and *J*). Dotted lines indicate the internal elastic lamina, which separates the intima from the media, and the external elastic lamina, which separates the media from the adventitia. Arrows in *G* and *H* and *I* and *J* denote Ki67-positive SMCs. Boxed regions in *I* and *J* are magnified views. H&E, hematoxylin-eosin; NRP1, neuropilin 1; SMA, smooth muscle actin; SMC, smooth muscle cell.

To determine the role of SMC-*Nrp1* in pathological SMC accumulation giving rise to neointima formation and vascular remodeling, we utilized the mouse perivascular cuff model of vessel injury, in which neointima formation is induced by placement of a nonocclusive cuff around the femoral artery, without removal of the endothelial cell layer (29). We detected a significant reduction in neointima formation in the *Nrp1^SMCiKO^* mutants versus controls at 21 days following cuff placement and tamoxifen treatment to reduce *Nrp1* expression in arterial SMC (Fig. 6, *A* and *C*–*F*). Cell proliferation in the media in *Nrp1^SMCiKO^* mice was decreased versus controls, as measured by ki67 staining (Fig. 6, *G*–*J*), and the reduction in proliferation was statistically significant (Fig. 6*B*), indicating that reduced SMC proliferation at least partly explains reduced neointima formation in *Nrp1^SMCiKO^* mice (24). It is likely that medial proliferation of the SMCs is followed by the migration of these cells through the internal elastic lamina into the intima. Studies have also implicated adventitial myofibroblasts in the development of neointima (33, 34). These results are consistent with inhibition of SMC/myofibroblast migration and proliferation following genetic ablation of *Nrp1* expression in these cells in vivo.

In conclusion, this study identifies a novel requirement for *Nrp1* in SMCs and myofibroblasts during alveolar development in vivo. A role for *Nrp1* in lung development is also supported by an earlier finding that constitutive loss of semaphorin-NRP1 signaling due to knock-in of mutant *Nrp1* unable to bind Sema3 ligands led to acute respiratory distress and high neonatal mortality, which was associated with loss of alveolar myofibroblasts at sites of presumptive septal tips (18), as we found in *Nrp1^SMCiKO^* mice. However, Joza et al. did not identify the cell types responsible for the phenotypes observed. Since manifestation of the phenotype waned later in postnatal life and later induction of knockout resulted in a milder phenotype, our findings indicate a time-specific requirement for *Nrp1*-expressing SMCs in the early stages of postnatal lung development, whereas *Nrp1* loss in SMCs at later stages of postnatal lung development may not be required or could be compensated by NRP2 and/or other mechanisms. This conclusion is consistent with the report that postnatal deletion of *Nrp1* at P5, using the tamoxifen-inducible *Esr1-Cre* transgene, only caused a mild, transient alveolar and vascular phenotype, indicating that expression of *Nrp1* after P5 is not essential for alveolar development or vascular function (19). Given that PDGF-A is essential for alveolar (septal) myofibroblast development and alveogenesis (7), the impairment of Nrp1-deficient pulmonary myofibroblast migration in response to PDGF-AA in vitro and the impairment of PDGF-BB-induced SMC outgrowth in *Nrp1^SMCiKO^* aortic rings suggest that *Nrp*1 may be important for PDGF signaling in both SMCs and myofibroblast migration and recruitment to septae during alveolar maturation. Our findings may have relevance for neonatal respiratory disorders such as bronchopulmonary dysplasia (BPD). Absence of alveolar myofibroblasts has been implicated in the pathology of BPD (2), and levels of VEGF and VEGFR1/R2 are decreased in BPD (8), whereas downregulation of *Nrp1*, *Vegfr1*, and *Vegfr2* was reported in a baboon model of BPD (40). Conversely, though alveolar myofibroblasts are abundant during alveolarization, they are absent in adult lungs except in fibrotic lung diseases such as interstitial fibrosis where they are implicated in disease pathogenesis (45). NRP1 may therefore be a therapeutic target in fibrotic diseases of the lung and other pathologies in which excessive SMC/myofibroblast proliferation plays a role.

Our findings demonstrate that SMC expression of *Nrp1* is largely dispensable for early postnatal vascular development in the retina and, since these mice appear normal and viable, is therefore seemingly not required for SMC maturation in arteriogenesis more generally. The apparent restriction of early postnatal defects in *Nrp1^SMCiKO^* to the lung may be due to the important role of NRP1 in myofibroblast recruitment during postnatal alveolar development, as revealed in this study; in contrast, myofibroblasts may be less essential for postnatal retinal vascularization or in development and expansion of other vascular beds.

Our results also demonstrate a requirement for *Nrp1* in pathological SMC/myofibroblast proliferation and neointima formation in vivo in a mouse perivascular cuff model, in agreement with our previous findings demonstrating inhibition of neointima formation due to targeted shRNA-mediated knockdown of *Nrp1* and *Nrp2* in the rat balloon carotid artery injury model (32). Taken together, these data support a role for *Nrp1* in pathological neointimal SMC remodeling in response to vascular injury, findings that may be relevant for vasculoproliferative diseases such as atherosclerosis and arterial stenosis following angioplasty and transplantation.

## GRANTS

This work was supported by British Heart Foundation program Grant RG/06/003.

## DISCLOSURES

No conflicts of interest, financial or otherwise, are declared by the authors.

## AUTHOR CONTRIBUTIONS

I.Z. conceived and designed research; M.M., I.M.E., V.M., C.P.-M., and K.P. performed experiments; M.M. and I.Z. analyzed data; M.M. and I.Z. interpreted results of experiments; M.M. and I.M.E. prepared figures; M.M. and I.Z. drafted manuscript; I.M.E. and I.Z. edited and revised manuscript; I.Z. approved final version of manuscript.
